# Optimal duration of antiviral treatment in patients with gastrointestinal cytomegalovirus disease at a low and high risk of relapse

**DOI:** 10.1097/MD.0000000000028359

**Published:** 2022-01-07

**Authors:** Kyung Hwa Jung, Jiwon Jung, Min Jae Kim, Yong Pil Chong, Sang-Oh Lee, Sang-Ho Choi, Yang Soo Kim, Sung-Han Kim

**Affiliations:** a Department of Infectious Diseases, Asan Medical Center, University of Ulsan College of Medicine, Seoul, Republic of Korea; b Department of Infectious Diseases, Uijeongbu Eulji Medical Center, University of Eulji College of Medicine, Uijeongbu, Republic of Korea.

**Keywords:** antiviral treatment, gastrointestinal cytomegalovirus disease, relapse

## Abstract

We evaluated the association between antiviral treatment duration and relapse of gastrointestinal (GI) cytomegalovirus (CMV) disease by analyzing the risk factors for relapse.

Patients who were diagnosed with GI CMV disease at a tertiary hospital from January 2008 to April 2019 were retrospectively enrolled. Patients with relapsed disease were those with a recurrence of GI CMV disease at least 4 weeks after the initial antiviral treatment.

Of 238 participants, including 145 (51.9%) with upper and 93 (48.1%) with lower GI CMV diseases, 27 (11.3%) had experienced relapses. The difference in antiviral treatment duration between the relapsed and nonrelapsed GI CMV groups was not significant (median days, 21.0 vs 17.0, *P* = .13). Multivariate analysis revealed that hematologic malignancy (odds ratio, 3.73; *P* = .026) and ulcerative colitis (odds ratio, 4.61; *P* = .003) were independent risk factors for relapse. Participants with at least one of these risk factors and those with no independent risk factors were classified under the high- (relapse rate, 25.9%) and low-risk of relapse groups (relapse rate, 6.7%), respectively. Accordingly, we further stratified 180 (75.6%) and 58 (24.4%) participants under the low- and high-risk of relapse groups, respectively. There was no significant difference in relapse rates between the high- and low-risk groups according to antiviral treatment duration.

Approximately 10% of the participants experienced relapses after antiviral treatment, with hematologic malignancy and ulcerative colitis featuring as risk factors. Therefore, prolonged antiviral treatment might not be helpful in preventing GI CMV disease relapse.

## 1 Introduction

Cytomegalovirus (CMV) is considered to be one of the most important pathogens in immunocompromised patients such as solid organ transplant (SOT) or bone marrow transplant recipients.^[1,2]^ Tissue-invasive CMV disease is characterized by predominant symptoms and localization to a specific tissue site.^[3]^ Gastrointestinal (GI) CMV disease is the most frequent form of tissue-invasive CMV diseases.^[4]^ However, some patients experienced relapses even after appropriate antiviral treatment. In previous studies, approximately 23% to 33% patients with primary CMV disease experienced relapses after antiviral treatment following SOT.^[5,6]^

In this context, clinicians tend to prolong the duration of use of antiviral agents due to the concern of relapse of CMV diseases; however, recent guidelines have recommended that the duration of antiviral treatment should be individualized based on the resolution of clinical symptoms and virologic clearance.^[7,8]^ However, there are limited studies on the appropriate duration of antiviral treatment based on the anatomic sites of CMV diseases. A previous study on endoscopic responders and nonresponders in patients with upper GI CMV disease showed that prolonged antiviral treatment (≥28 days) was not an independent risk factor for CMV disease relapse.^[9]^ Therefore, unlike in the case of CMV retinitis, some authors reported that the majority of patients who received antiviral agents for 2 to 4 weeks showed favorable clinical responses.^[10]^ In contrast, some experts still recommended a 2 to 3 week-long induction therapy, followed by several weeks of maintenance therapy.^[11]^ We thus evaluated the association between the duration of antiviral treatment and the relapse of GI CMV disease by analyzing the risk factors for the relapse of GI CMV disease.

## 2 Methods

### 2.1 Study population

In this retrospective cohort study, patients aged >18 years diagnosed with upper or lower GI CMV disease who were admitted to the Asan Medical Center, Seoul, South Korea, a tertiary care teaching hospital, between January 2008 and April 2019 were enrolled (n = 249). The patients who were not treated for GI CMV diseases (n = 9) or aged <18 years (n = 2) were excluded. Data on the following parameters were collected: age, sex, symptoms and signs at the time of diagnosis, underlying diseases, sites of involvement in the GI tract, endoscopic findings, clinical outcomes, and relapse status. All the patients diagnosed with GI CMV disease received antiviral treatment with agents such as ganciclovir or valganciclovir. Antiviral treatment was administered at least until the resolution of clinical symptoms and CMV antigenemia or until negative results were obtained for polymerase chain reaction (PCR) tests using blood or biopsy tissue. CMV antigenemia and PCR tests were performed as described elsewhere.^[12,13]^ The study protocol was approved by the institutional review board of Asan Medical Center (2020-0104). Informed consent was waived because of the retrospective nature of this study.

### 2.2 Definitions

GI CMV disease was categorized as a “proven CMV GI disease,”, “probable CMV GI disease”, and “possible CMV GI disease” based on some modifications of the recent Infectious Diseases Society of America guidelines.^[3]^ “Proven GI CMV disease” was defined as the presence of lower GI symptoms, macroscopic mucosal lesions, and CMV documented in the tissue via histopathology or immunohistochemistry. “Probable GI CMV disease” was defined as the presence of lower GI symptoms and CMV documented in the tissue via histopathology or immunohistochemistry and the absence of macroscopic mucosal lesions. “Possible GI CMV disease” was defined as the presence of CMV documented via PCR testing from tissue biopsies. “Relapsed GI CMV disease” was defined as a previously documented GI CMV disease at least 4 weeks after completion of the initial antiviral treatment and a new diagnosis of GI CMV disease.^[3]^ Immunocompromised patients were defined as patients with underlying diseases, such as human immunodeficiency virus infections, malignancies, liver cirrhosis, and chronic renal failure, undergoing immunosuppressive treatment or steroid therapy.^[14]^

### 2.3 Statistical analyses

Data are expressed as the medians and inter quartile ranges (IQR) or means ± standard deviations. Continuous data were compared using Student *t* test or Mann–Whitney *U* test if the distribution was variable. Categorical data were described using contingency tables and a chi-squared test or Fisher exact test. A univariate analysis was performed using logistic regression to determine the risk factors independently associated with a relapse of gastrointestinal CMV disease. Subsequently, multiple logistic regression analysis was performed for variables with a *P-*value <.2 in the univariate analysis, based on the backward Elimination (Wald) method. The results are reported as odds ratios (ORs) with a 95% confidence interval (CI). A *P-*value <.05 was considered significant. The calculations were performed using SPSS for Windows software package version 21.0 (IBM Co., Armonk, NY).

## 3 Results

### 3.1 Patient characteristics and clinical outcomes

**Figure 1:**
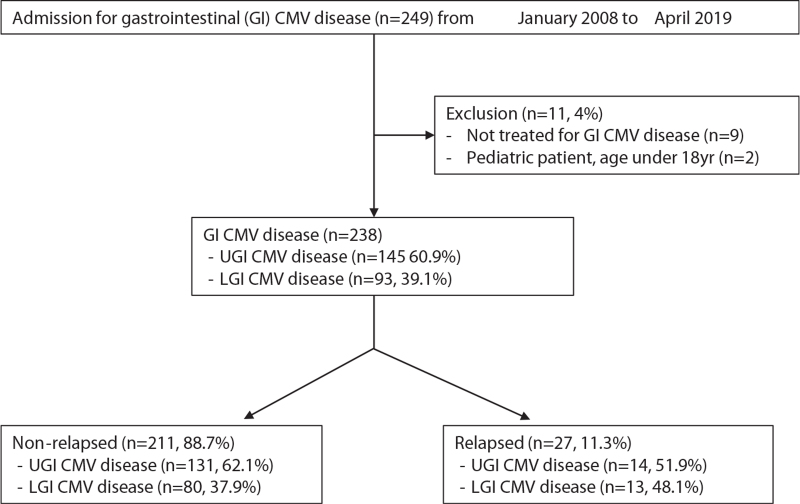
Flowchart of study inclusion.

A total of 249 patients who were admitted and diagnosed with GI CMV disease were retrospectively analyzed. Eleven of them were excluded due to the following reasons: they were observed without administering antiviral treatment for GI CMV disease (n = 9) and they were pediatric patients aged <18 years (n = 2). Finally, 238 patients were included in this study (Fig. 1).

The clinical characteristics and outcomes of the patients are shown in Table 1. Among these patients, 145 (51.9%) and 93 (48.1%) were diagnosed with upper and lower GI CMV disease, respectively. Median duration of antiviral treatment, days (IQR) were 18.0 (14.0–25.5). Out of these, 27 (11.3%) had experienced relapse of the disease. Median follow-up days (IQR) in nonrelapsed group were 257 (102–1129), and in relapsed group were 452 (319.8-632), respectively. Median time to relapse, days (IQR) were 127.0 (32.0–261). Among the initial clinical symptoms and signs, hematochezia and melena were significantly more frequent in the relapsed group than in the nonrelapsed group (51.9% vs 27.0%; *P* = .008). There were no statistical differences in the underlying diseases except for ulcerative colitis (UC) (relapsed group 37.0% vs nonrelapsed group 10.9; *P* = .001). There was no statistical difference between the 2 groups in the rate of relapse with respect to immune status. Additionally, there was no significant difference in the duration of antiviral treatment between the relapsed group and the nonrelapsed group (median 21.0 days and 17.0 days, *P* = .13) (Table 1).

**Table 1:** Clinical characteristics and outcomes in patients with nonrelapsed and relapsed GI CMV disease.

Variable	Total (n = 238)	Nonrelapsed (n = 211)	Relapsed (n = 27)	*P-*value
Age, median (IQR), yr	59 (48–67)	59 (48–64)	59 (51–65)	.943
Male gender (%)	149 (62.6)	131 (62.1)	18 (12.1)	.643
Initial clinical symptoms and signs (%)				
Fever or chills	22 (9.2)	20 (9.5)	2 (7.4)	1.000
Nausea or vomiting	30 (12.6)	26 (12.3)	4 (14.8)	.757
Hematochezia or melena	71 (29.8)	57 (27.0)	14 (51.9)	.008
Diarrhea	54 (22.7)	49 (23.2)	5 (18.5)	.583
Underlying disease/procedure (%)				
Diabetes mellitus	55 (23.1)	49 (23.2)	6 (22.2)	.908
Ulcerative colitis	33 (13.9)	23 (10.9)	10 (37.0)	.001
Crohn disease	4 (1.7)	3 (1.4)	1 (3.7)	.384
Others^∗^	37 (15.5)	35 (16.6)	2 (7.4)	.215
Immunocompetent host	65 (27.3)	56 (86.2)	9 (13.8)	.456
Immunocompromised host^†^ (%)	173 (72.7)	155 (73.5)	18 (66.7)	.456
Solid tumor	30 (12.6)	27 (12.8)	3 (11.1)	1.000
Hematologic malignancy	25 (10.5)	20 (9.5)	5 (18.5)	.176
Transplantation	108 (45.4)	99 (46.9)	9 (33.3)	.182
Solid organ	100 (42.0)	92 (43.6)	8 (29.6)	.166
Hematopoietic stem cell	10 (4.2)	9 (4.3)	1 (3.7)	.891
Chronic kidney disease	22 (9.2)	20 (9.5)	2 (7.4)	1.000
Liver cirrhosis	9 (3.8)	9 (4.3)	0	.603
HIV infection	7 (2.9)	7 (3.3)	0	1.000
Medication before the diagnosis of GI CMV disease (%)				
Steroid use^‡^	147 (61.8)	129 (61.1)	18 (66.7)	.578
Immunosuppressant use^§^	151 (63.4)	134 (63.5)	17 (63.0)	1.000
Treatment of acute rejection (%)	9/108 (8.3)	9/99^∗∗^ (9.1)	0/9 (0)	1.000
CMV prophylaxis^||^ (%)	37/108 (34.3)	32/99 (32.3)	5/9 (55.6)	.269
Upper GI CMV disease (%)	145 (60.9)	131 (62.1)	14 (51.9)	.305
Lower GI CMV disease (%)	93 (39.1)	80 (37.9)	13 (48.1)	.305
GI CMV disease (%)				
Proven^¶^	195 (81.9)	173 (82.0)	22 (81.5)	1.000
Probable^#^	19 (8.0)	17 (8.1)	2 (7.4)	1.000
Possible^∗∗^	24 (10.1)	21 (10.0)	3 (11.1)	.742
Initial antiviral therapy (%)				
Ganciclovir	236 (99.2)	209 (99.1)	27 (100)	1.000
Valganciclovir	21 (8.8)	19 (9.0)	2 (7.4)	1.000
Median duration of antiviral treatment, (IQR)	18.0 (14.0–25.5)	17.0 (14.0–27.0)	21.0 (16.0–22.0)	.125
Median time to negative CMV viremia,^††^ d (IQR)	15.0 (11.0–21.0)(n = 120)	15.0 (10.3–21.0)(n = 104)	15.5 (13.3–21.8)(n = 16)	.583
Median time to relapse, d (IQR)	N/A	N/A	127.0 (32.0–261.0)	N/A
Mortality (%)				
In-hospital mortality	21 (8.8)	20 (9.5)	1 (3.7)	.482
30-d mortality	9 (3.8)	9 (6.5)	0	.604
60-d mortality	14 (5.9)	14 (10.1)	0	.219
90-d mortality	18 (7.6)	16 (11.6)	2 (10.0)	1.000
Cause of death (%)				
CMV colitis-related	1/21 (4.8)	1/20 (5.0)	0	1.000
Uncertain	1 /21 (4.8)	1/20 (5.0)	0	1.000
Not related	19/21 (90.5)	18/20 (90.0)	1/1 (100)	1.000

### 3.2 Risk factors associated with the relapse of GI CMV disease

The risk factors associated with the relapse of GI CMV disease are shown in Table 2. In the univariate analysis, the risk factors were the presentation of hematochezia and melena as the initial clinical symptoms and signs, and hematologic malignancy, SOT, and UC as underlying diseases. In the multivariate analysis, hematologic malignancy (OR, 3.73; 95% CI, 1.17–11.86; *P* = .026) and UC (OR, 4.61; 95% CI, 1.70–12.49; *P* = .003) were independent risk factors of relapse of GI CMV disease.

**Table 2:** Univariate and multivariate analyses of the risk factors of relapse of gastrointestinal cytomegalovirus disease.

	Univariate analysis		Multivariate analysis	
Characteristics	Unadjusted OR (95% CI)	*P*	Adjusted OR (95% CI)	*P*
Initial clinical symptom or sign				
Hematochezia or melena	2.91 (1.29–6.57)	.010		
Immunocompetent host	0.72 (0.31–1.70)	.456		
Underlying disease				
Hematologic malignancy	2.17 (0.74–6.36)	.158	3.73 (1.17–11.86)	.026
Solid organ transplantation	0.54 (0.23–1.30)	.171		
Ulcerative colitis	4.81 (1.97–11.75)	.001	4.61 (1.70–12.49)	.003
Duration of antiviral treatment	1.00 (0.98–1.02)	.911		

### 3.3 Relapse rate according to the total duration of antiviral treatment

According to the results of the multivariate analysis, we further classified patients into those with high and low risks of relapse. The high-risk group included patients with at least one of the 2 risk factors for relapse (relapse rate, 25.9%). Patients without these 2 risk factors were included in the low-risk group (relapse rate, 6.7%). According to this definition, 180 (75.6%) and 58 (24.4%) patients were in the low-risk and high-risk groups of relapse of CMV disease, respectively. Subsequently, no significant difference was observed in the rate of relapse between the groups according to the duration of antiviral treatment in the high-risk and low-risk groups (Figure S1, Supplemental Digital Content). The patient characteristics and clinical outcomes of the 238 patients with GI CMV disease according to the duration of antiviral treatment are shown in Table S1, Supplemental Digital Content. Relapse rates of patients with GI CMV disease in immunocompetent host and immunocompromised host according to the total duration of antiviral treatment were shown in Figure S2 (Supplemental Digital Content).

## 4 Discussion

Previous studies have shown that the relapse rate of the first episode of CMV disease after a defined course of antiviral treatment in patients with SOT or bone marrow transplantation ranged from 23% to 33%.^[5,6,15]^ In addition, Sia et al^[5]^ and Humar et al^[6]^ reported that the relapse rates of CMV disease were 12.5% (1/8) and 21% (8/24), respectively. In patients with human immunodeficiency virus, the relapse rate of CMV antigenemia or GI CMV disease was reported to be 39% (7/18).^[16]^ The variations in relapse rate may be attributed to the heterogenicity in host factors and the type of tissue-invasive CMV disease. Generally, prolonged antiviral treatment is a preferred option for CMV retinitis, whereas a shortened antiviral treatment is suggested for GI CMV diseases. The relapse rate of CMV diseases was not significantly associated with the duration of antiviral treatment.^[17–19]^ Eid et al^[17]^ reported that the relapse rate of GI CMV disease in patients with SOTs was not significantly associated with a longer duration of induction antiviral therapy and the administration of maintenance therapy. Asberg et al^[20]^ reported that the relapse rates of CMV diseases, including GI CMV disease, were similar between patients who received and did not receive maintenance valganciclovir therapy. Therefore, these studies critically question whether maintenance therapy may prove to be beneficial in the reduction of relapse rate, especially in GI CMV disease. Our large cohort study on GI CMV disease strengthened this concept by demonstrating that a prolonged antiviral treatment was not associated with the relapse of GI CMV disease. Rapid renewal of the GI epithelium and a relatively high concentration of ganciclovir in the GI mucosa compared to that in the retinal tissue might favor a relatively shortened antiviral treatment in patients with GI CMV disease.

The risk factors of the relapse of CMV disease were the extent of CMV disease, persistent CMV deoxyribonucleic acidemia at the end of induction treatment (day 21), lung transplantation, CMV donor seropositive and recipient seronegative status, and a history of recent acute rejection treatment.^[5,6,17,18,21,22]^ In immunocompetent patients, a critically ill status may be a major risk factor of tissue-invasive CMV disease.^[23]^ In patients with cancer, male sex, low body mass index, lymphopenia, hematologic malignancy, steroid use, and red blood cell transfusion in the past month were identified as independent risk factors of GI CMV disease.^[24]^ In our study population including 25% of immunocompetent patients, the overall relapse rate of GI CMV disease was higher in the high-risk group (relapse rate, 25.9%) than in the low-risk group (relapse rate, 6.7%); the high-risk group included patients with at least one of the 2 risk factors, such as hematologic malignancy and UC, according to the multivariate analysis. Clinicians usually prefer a more prolonged antiviral treatment in patients with a high risk for the relapse of GI CMV disease. We thus performed an analysis stratified by relapse risk according to the duration of antiviral treatment. This stratified analysis also revealed that the duration of antiviral treatment was not associated with the relapse rate of GI CMV diseases.

Our study had several limitations. First, since it was retrospectively performed in a large tertiary referral center, there could be a selection bias. Therefore, a well-designed prospective multi-center cohort study could provide more valuable information on the association between an optimal duration of antiviral treatment and relapse of GI CMV disease. Second, the patient population in this study was not homogenous in terms of underlying diseases and immune statuses. Underlying diseases and immunosuppression may affect the relapse rate of GI CMV disease according to duration of antiviral treatment. Moreover, the heterogenicity of the underlying diseases and immune statuses may have a reduced statistical power. Despite the heterogenicity in the underlying diseases, our study included a relatively large number of immunocompetent patients, which may explain the overall low relapse rate. Therefore, this study may significantly contribute to the literature related to our understanding on GI CMV diseases in immunocompetent patients and the necessity of prolonged antiviral treatment in GI CMV disease. Third, we could not assess CMV-specific T-cell responses. Recently, it has been reported that the cell-mediated immune response to CMV may predict the relapse of CMV diseases.^[25]^ Finally, considering high frequency of CMV seropositivity (>95%) of Korean adults, the majority of infection ways in our study were more likely to be reactivated CMV infection rather than primary CMV infection.^[26]^ So, further studies are needed in various CMV prevalence setting and more specific markers to predict relapse and to customize antiviral treatment.

In conclusion, approximately 10% of the patients with GI CMV disease experienced relapses after antiviral treatment and these outcomes were common in those with hematologic malignancy and UC. Our data suggested that prolonged antiviral treatment might not be helpful in preventing the relapse of GI CMV disease (Figure S2, Supplemental Digital Content).

## Author contributions

**Conceptualization:** Sung-Han Kim.

**Data curation:** Kyung Hwa Jung.

**Formal analysis:** Kyung Hwa Jung.

**Investigation:** Jiwon Jung.

**Methodology:** Sung-Han Kim.

**Project administration:** Sung-Han Kim.

**Supervision:** Sung-Han Kim, Jiwon Jung, Min Jae Kim, Yong Pil Chong, Sang-Oh Lee, Sang-Ho Choi, Yang Soo Kim.

**Writing – original draft:** Kyung Hwa Jung.

**Writing – review & editing:** Sung-Han Kim.

## Supplementary Material

Supplemental Digital Content

## Supplementary Material

Supplemental Digital Content

## Supplementary Material

Supplemental Digital Content

## Supplementary Material

Supplemental Digital Content
