# Supplementary material for: Optimal duration of antiviral treatment in patients with gastrointestinal cytomegalovirus disease at a low and high risk of relapse
Source: Medicine (Baltimore). 2022 Jan 7;101(1):e28359. doi: 10.1097/MD.0000000000028359 (PMC8735784; doi:10.1097/MD.0000000000028359)
Supplement: Supplemental Digital Content [file medi-101-e28359-s002.docx]

**Supplementary Table 1. Baseline clinical characteristics and clinical outcomes of the 238 patients with gastrointestinal cytomegalovirus disease and treated with antiviral agents**

| **Variable** | **Duration of Antiviral treatment** | | | | ***p*** |
| --- | --- | --- | --- | --- | --- |
|  | **Under 2 weeks**  **(n = 71)** | **≤2 ~ <3 weeks**  **(n = 84)** | **≤3 ~ <4 weeks**  **(n = 36)** | **Over 4 weeks**  **(n = 47)** |  |
| Age, median (IQR), years | 61 (51–73) | 56.5 (47.3–65) | 59.5 (46.8–66.8) | 59.0 (51.0–65.0) | 0.140 |
| Male gender (%) | 43 (60.6) | 54 (64.3) | 23 (63.9) | 29 (61.7) | 0.966 |
| Initial clinical symptom or sign (%) |  |  |  |  |  |
| Fever or chills | 7 (9.9) | 10 (11.9) | 0 | 5 (10.6) | N/A |
| Nausea or vomiting | 12 (16.9) | 8 (9.5) | 4 (11.1) | 6 (12.8) | 0.579 |
| Hematochezia or melena | 18 (25.4) | 26 (31.0) | 16 (44.4) | 11 (23.4) | 0.197 |
| Diarrhea | 18 (25.4) | 14 (16.7) | 8 (22.2) | 14 (29.8) | 0.339 |
| Underlying disease/procedure (%) |  |  |  |  |  |
| Diabetes mellitus | 18 (25.4) | 22 (26.2) | 6 (16.7) | 9 (19.1) | 0.564 |
| Ulcerative colitis | 9 (12.7) | 13 (15.5) | 9 (25.0) | 2 (4.3) | 0.022 |
| Crohn’s disease | 1 (1.4) | 2 (2.4) | 1 (2.8) | 0 | 0.722 |
| Chronic obstructive lung disease | 1 (1.4) | 3 (3.6) | 3 (8.3) | 1 (2.1) | 0.492 |
| Heart failure | 2 (2.8) | 1 (1.2) | 2 (5.6) | 3 (6.4) | 0.416 |
| Rheumatologic disease | 3 (4.2) | 2 (2.4) | 2 (5.6) | 3 (6.4) | 0.707 |
| Immunocompetent host | 25 (38.5) | 27 (41.5) | 7 (10.8) | 6 (9.2) | 0.001 |
| Immunocompromised host^a^ | 46 (26.6) | 57 (32.9) | 29 (16.8) | 41 (23.7) | 0.010 |
| Solid tumor | 14 (19.7) | 9 (10.7) | 3 (8.3) | 4 (8.5) | 0.270 |
| Hematologic malignancy | 4 (5.6) | 9 (10.7) | 5 (12.9) | 7 (14.9) | 0.308 |
| Transplantation | 25 (35.2) | 36 (42.9) | 17 (47.2) | 30 (63.8) | 0.021 |
| Solid organ | 25 (35.2) | 35 (41.7) | 15 (41.7) | 25 (53.2) | 0.291 |
| Hematopoietic stem cell | 0 | 3 (3.6) | 2 (5.6) | 5 (10.6) | N/A |
| Chronic kidney disease | 6 (8.5) | 8 (9.5) | 1 (2.8) | 7 (14.9) | 0.159 |
| Liver cirrhosis | 3 (4.2) | 4 (4.8) | 0 | 2 (4.3) | 0.640 |
| HIV | 0 | 5 (6.0) | 0 | 2 (4.3) | N/A |
| Medication before the diagnosis of GI CMV disease (%) |  |  |  |  |  |
| Steroid use^b^ | 36 (50.7) | 51 (60.7) | 24 (66.7) | 36 (76.6) | 0.030 |
| Immunosuppressant use^c^ | 34 (47.9) | 56 (65.5) | 26 (72.2) | 36 (76.6) | 0.008 |
| Treatment of acute rejection | 5/25^i^ (20.0) | 0 | 0 | 4/30 (13.3) | N/A |
| Treatment of CMV prophylaxis^d^ | 6/25 (24.0) | 6/36 (16.7) | 6/17 (35.3) | 19/30 (63.3) | 0.001 |
| Upper GI CMV disease | 42 (59.2) | 52 (61.9) | 20 (55.6) | 31 (66.0) | 0.789 |
| Lower GI CMV disease | 29 (40.8) | 32 (38.1) | 16 (44.4) | 16 (34.0) | 0.789 |
| CMV colitis |  |  |  |  |  |
| Proven^e^ | 54 (76.1) | 70 (83.3) | 33 (91.7) | 38 (80.9) | 0.155 |
| Probable^f^ | 5 (7.0) | 6 (7.1) | 2 (5.6) | 6 (12.8) | 0.594 |
| Possible^g^ | 12 (16.9) | 8 (9.5) | 1 (2.8) | 3 (6.4) | 0.058 |
| Initial antiviral therapy |  |  |  |  |  |
| Ganciclovir | 70 (98.6) | 84 (100) | 35 (97.2) | 47 (100) | N/A |
| Valganciclovir | 6 (8.5) | 4 (4.8) | 2 (5.6) | 9 (19.1) | 0.142 |
| Median duration of antiviral treatment, (IQR) | 14.0 (12.0–14.0) | 17.0 (15.0–20.8) | 23.0 (22.0–22.8) | 40.0 (31.0–55.0) | <0.001 |
| Median time to negative CMV viremia^h^ in days (IQR) | 11.0 (7.0–14.0) | 15.0 (13.0–19.0) | 16.0(13.0–21.0) | 20.0 (11.0–28.0) | 0.160 |
| Outcomes |  |  |  |  |  |
| Relapse after initial treatment | 4 (5.6) | 12 (14.1) | 7 (19.4) | 4 (8.5) | 0.134 |
| Immunocompetent host | 2 (22.2) | 4 (44.4) | 3 (33.3) | 0 (0) | 0.250 |
| Immunocompromised host | 2 (11.1) | 8 (44.4) | 4 (22.2) | 4 (22.2) | 0.217 |
| Median time to relapse, days (IQR) | 45.0 (19.5–373.0) | 156.5 (42.3–242.0) | 192.0 (30.0–2222.0) | 89.0 (45.0–137.5) | 0.204 |
| Mortality |  |  |  |  |  |
| In-hospital mortality | 9 (12.7) | 5 (5.9) | 1 (2.8) | 6 (12.8) | 0.129 |
| 30-day mortality | 5 (8.9) | 2 (2.4) | 1 (2.8) | 1 (3.6) | 0.568 |
| 60-day mortality | 8 (14.3) | 3 (3.5) | 1 (2.8) | 2 (7.1) | 0.294 |
| 90-day mortality | 8 (14.3) | 5 (5.9) | 1 (2.8) | 4 (8.5) | 0.313 |
| Cause of death |  |  |  |  |  |
| CMV colitis-related | 0 | 0 | 1/1 (100) | 0 | N/A |
| Uncertain | 0 | 1/5 (20.0) | 0 | 0 | N/A |
| Not related | 9/9 (100) | 4/5 (80.0) | 0 | 6/6 (100) | N/A |

Data are presented as numbers (%) unless otherwise indicated.

Abbreviations: IQR, interquartile range; HIV, human immunodeficiency virus; GI CMV, gastrointestinal cytomegalovirus; IHC, immunohistochemistry; PCR, polymerase chain reaction

^a^An immunocompromised host was defined as a patient with an underlying disease such as a human immunodeficiency virus infection, malignancy, liver cirrhosis, or chronic renal failure or as one who was receiving immunosuppressive treatment or corticosteroid treatment.

^b^Corticosteroid use is defined as the use of corticosteroids at a mean minimum dose of 0.3 mg/kg/d of a prednisolone equivalent for ≥3 weeks.

^c^Treatment with immunosuppressants (e.g., tacrolimus, cyclosporine, sirolimus, azathioprine, or mycophenolate mofetil) in the last 90 days.

^d^Treatment of CMV prophylaxis was defined as primary prophylaxis for prevention of CMV disease in solid organ transplantation or hematopoietic transplantation patients.

^e^Proven CMV colitis was defined as gastrointestinal symptoms plus macroscopic mucosal lesions plus CMV documented in tissue by histopathology or immunohistochemistry.

^f^Probable CMV colitis was defined as gastrointestinal symptoms and CMV documented in tissue but without macroscopic mucosal lesions.

^g^Possible CMV colitis was defined as CMV documented in blood by a PCR test or antigenemia or CMV documented by PCR test from tissue biopsies.

^h^CMV viremia was defined as CMV documented by a PCR test or CMV antigenemia.

^i^Number of patients with a positive test result/number of patients tested
