# Supplementary material for: Optimal duration of antiviral treatment in patients with gastrointestinal cytomegalovirus disease at a low and high risk of relapse
Source: Medicine (Baltimore). 2022 Jan 7;101(1):e28359. doi: 10.1097/MD.0000000000028359 (PMC8735784; doi:10.1097/MD.0000000000028359)
Supplement: Supplemental Digital Content [file medi-101-e28359-s003.pptx]

## Slide 1
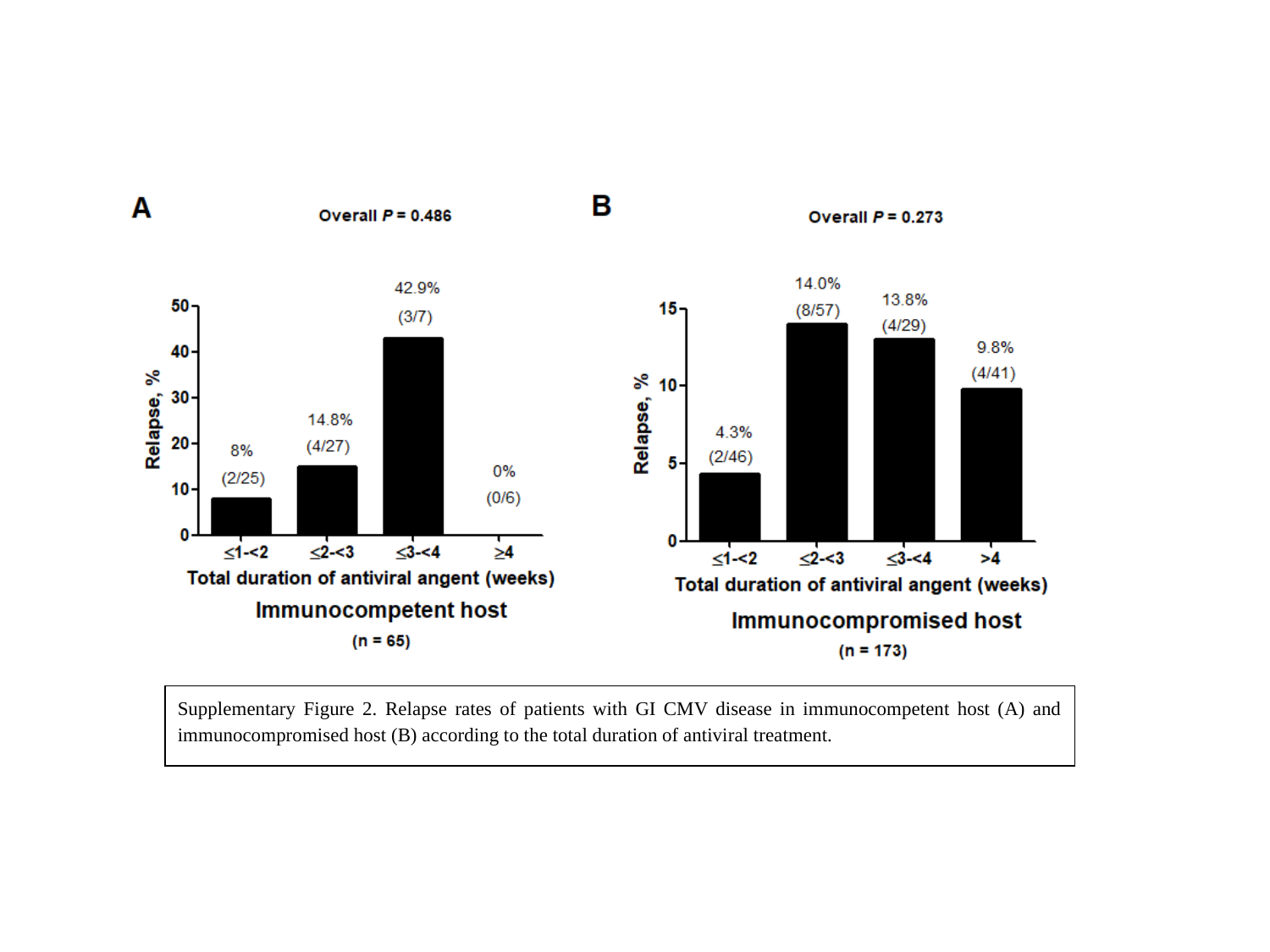

Supplementary Figure 2. Relapse rates of patients with GI CMV disease in immunocompetent host (A) and immunocompromised host (B) according to the total duration of antiviral treatment.
